# Severe hepatitis E virus genotype 3b in a patient with alcohol‑associated liver disease: A case report

**DOI:** 10.3892/mi.2024.146

**Published:** 2024-03-07

**Authors:** Tatsuo Kanda, Shuhei Arima, Reina Sasaki-Tanaka, Mai Totsuka, Masayuki Honda, Ryota Masuzaki, Naoki Matsumoto, Masahiro Ogawa, Masaharu Takahashi, Hiroaki Okamoto, Hirofumi Kogure

**Affiliations:** 1 Division of Gastroenterology and Hepatology, Department of Medicine, Nihon University School of Medicine, Tokyo 173-8610, Japan; 2 Division of Virology, Department of Infection and Immunity, Jichi Medical University School of Medicine, Shimotsuke, Tochigi 329-0498, Japan

**Keywords:** acute-on-chronic liver failure, alcohol, decompensated cirrhosis, hepatitis E virus genotype 3b, horse sashimi

## Abstract

Hepatitis E virus (HEV) infection occasionally causes acute-on-chronic liver failure in patients with alcohol-associated cirrhosis. These reports have been published mainly from highly HEV genotype 1-endemic countries. The present study describes the case of a patient with severe HEV genotype 3b infection and alcohol-associated liver disease. A male patient in his 70s who consumed alcohol, and who had begun consuming alcohol at the age of 12, had high levels of alanine aminotransferase (ALT) and total bilirubin. The peak levels of ALT and total bilirubin were 1,067 IU/l and 26.3 mg/dl, respectively. A computed tomography scan revealed an atrophic liver. Upon admission, both anti-HEV immunoglobulin A and HEV RNA were positive, and his HEV was genotype 3b. He also had chronic kidney disease, as his estimated glomerular filtration rate was <45 ml/min/1.73 m^2^, and ribavirin could not be used. The abnormal levels of the liver function parameters of the patient gradually improved due to conservative treatment, and he was discharged on day 43. On the whole, the present study demonstrates that careful attention should be paid to patients with viral hepatitis, including hepatitis E, when alcohol-associated liver disease is present. Novel anti-HEV drugs need to be developed for severe HEV infections with chronic kidney disease.

## Introduction

Hepatitis E virus (HEV) infection occurs in an estimated 20 million individuals, leading to an estimated 3.3 million symptomatic cases in developing and developed countries worldwide (1). Infection by HEV genotypes 1 and 2, which infect humans via the fecal-oral routes through the intake of contaminated foods and water, has mainly been reported in Southeast Asia and Mexico (2-5). HEV genotypes 3 and 4 are associated with zoonotic infection and are observed worldwide (6-9).

Acute hepatic insult manifesting as jaundice and coagulopathy, and complicated within 4 weeks by ascites and/or encephalopathy in patients with previously diagnosed or undiagnosed chronic liver disease is defined as acute-on-chronic liver failure (ACLF) (10). HEV genotype 1 plays a crucial role in acute viral hepatitis and ACLF in developing countries (11). Acute HEV genotype 1 infection is a leading cause of ACLF in Bangladesh and India (12,13).

HEV genotype 3 can also induce chronic infection in immunocompromised individuals and ACLF in patients with underlying liver disease (14-16). It has been reported that patients with HEV genotype 3 or 4 are susceptible to treatment with ribavirin (16).

Recently, the authors treated a Japanese male patient with severe acute HEV genotype 3b infection and alcohol-associated liver disease. The present study reports this case and discusses the possibility of ACLF induced by HEV genotype 3 infection and its treatment.

## Case report

A patient male in his 70s who consumed alcohol experienced abdominal distention, loss of appetite, epigastric pain and dark urine (jaundice). After 5 days, he visited the local clinic near his residence, and the worsening of his liver function was observed by the obtained test results. The following day, he was referred and admitted to Nihon University Itabashi Hospital (Tokyo, Japan).

Due to the patient's history of cerebral infarction, hypertension, diabetes mellitus and hyperuricemia, he regularly visited the local clinic. Aspirin, valsartan, amlodipine besylate, furosemide, sitagliptin phosphate hydrate, ipragliflozin L-proline, febuxostat and magnesium oxide were prescribed. He had also undergone surgery for his springer finger, and cefaclor, loxoprofen sodium salt and lebamipide were prescribed. He began to consume alcohol at 12 years of age. He had also consumed horse sashimi 1 month prior. He had no history of transfusion, tattooing, drug abuse or drug allergies, and had not recently traveled abroad. He had no family history of liver disease.

The height of the patient was 161 cm and his body weight was 69 kg. His blood pressure, pulse rate and body temperature were 157/93 mmHg, 71/min and 36.1˚C, respectively. A physical examination revealed that he was conscious; he had hepatic encephalopathy grade <2, and he had conjunctival icterus. Abdominal distention was observed.

The laboratory data of the patient obtained upon admission are presented in Table I, indicating marked liver dysfunction and a history of hepatitis B virus (HBV) infection. At 2 weeks following admission, positivity for anti-HEV immunoglobulin A (IgA) antibody (Institute of Immunology, Co. Ltd., Tokyo, Japan) was revealed. The patient was found to be HEV RNA-positive and he had acute HEV genotype 3b infection, as determined according to previously described methods (17). Furthermore, using stored serial serum samples, the IgG, IgM and IgA classes of HEV antibodies were determined as previously described (18), and all these HEV antibodies tested positive until the end of the observation period (day 83) (Table II). He also had chronic kidney disease with a severely decreased estimated glomerular filtration rate (category G4) (19) and type 2 diabetes mellitus.

**Figure 1:**
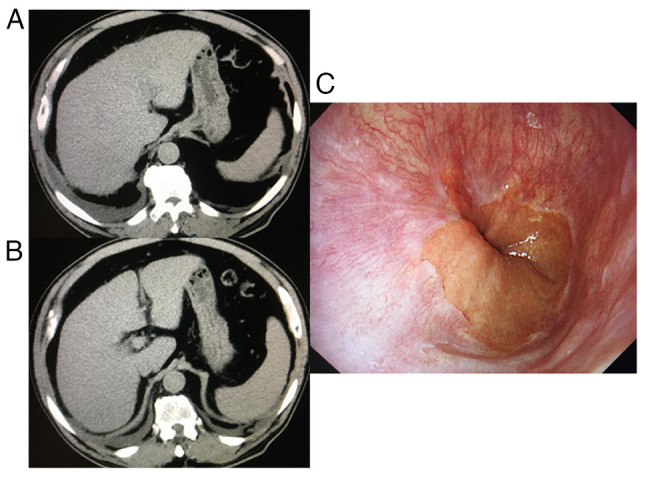
Abdominal CT scan and upper gastrointestinal endoscopic images. (A and B) Abdominal CT scan indicating signs of liver cirrhosis with paraumbilical vein dilatation, mild splenomegaly and right pleural effusion. (C) An upper gastrointestinal endoscopic examination did not reveal any esophageal varices. CT, computed tomography.

An abdominal computed tomography scan indicated an atrophic liver and cirrhosis, although no gastrointestinal varices were present, according to the endoscopy (Fig. 1). An abdominal ultrasound sonography revealed collateral veins and splenomegaly; his liver stiffness was 41.2 kPa according to transient elastography, indicating liver cirrhosis and inflammation, although he never experienced any episodes of ascites, jaundice, hepatic encephalopathy, or variceal bleeding (20). The patient was diagnosed with severe acute HEV genotype 3b and alcohol-associated liver cirrhosis.

**Figure 2:**
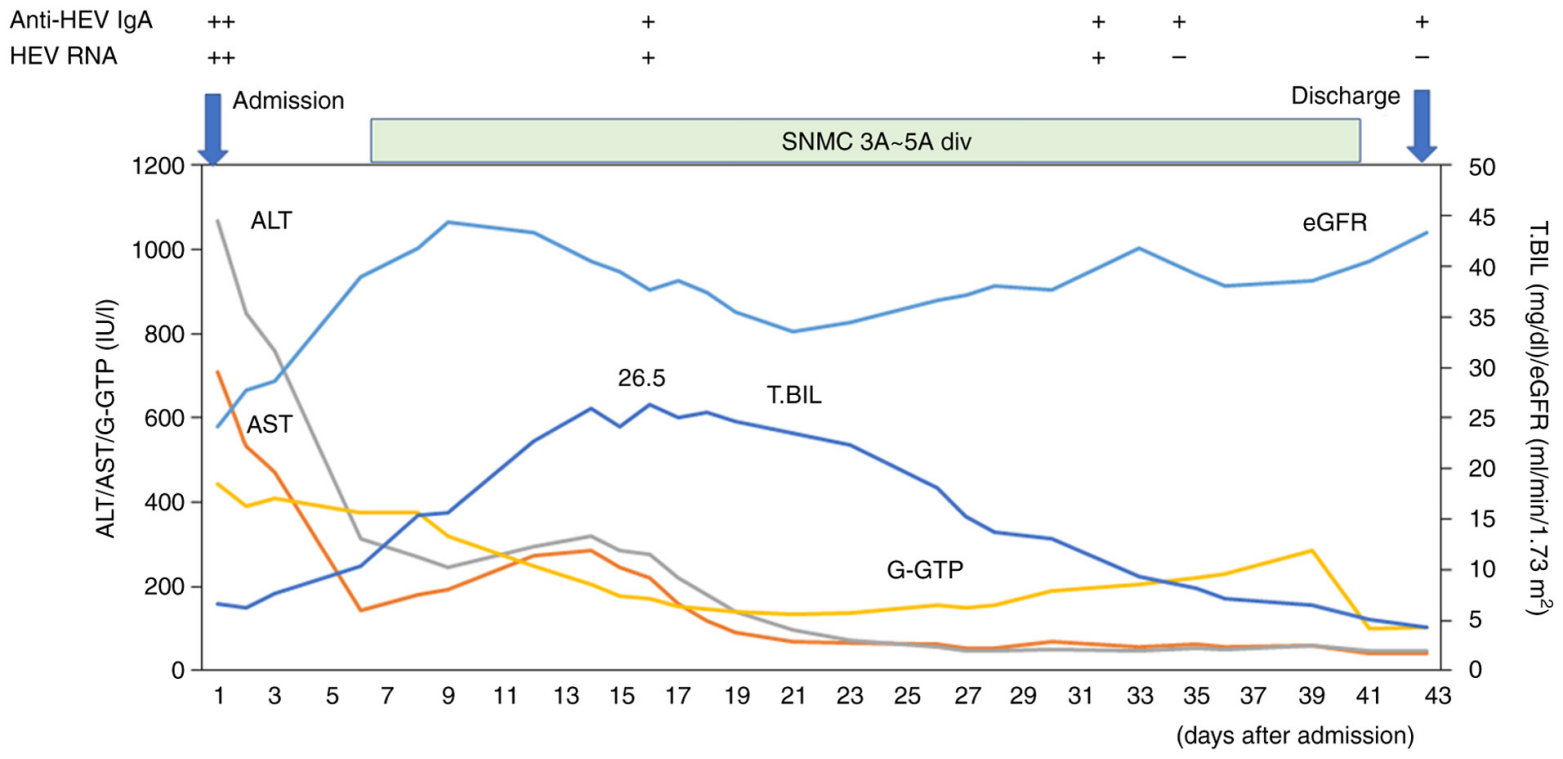
Clinical course of the patient in the present study. AST, aspartate aminotransferase; ALT, alanine aminotransferase; γ-GTP, γ-glutamyl transpeptidase; T. Bil, total bilirubin; eGFR, estimated glomerular filtration rate; anti-HEV IgA, anti-hepatitis E virus antibody immunoglobulin A; SNMC, Stronger Neo-Minophagen C with glycyrrhizin-containing preparation.

Due to his alcohol consumption shortly prior, indications for liver transplantation were not assessed. As ribavirin could not be used due to renal dysfunction (21), only conservative treatment was administered. However, his abnormal liver function tests gradually improved, although his HEV RNA was detectable by the highly sensitive nested reverse transcription-polymerase chain reaction with primers targeting the ORF2/ORF3 overlapping region (22) until day 32 after admission. The peak alanine aminotransferase (ALT) and total bilirubin levels were 1,067 IU/l and 26.3 mg/dl, respectively. He was ultimately discharged, and he left the hospital on foot on day 43 (Table II and Fig. 2).

## Discussion

Barbosa *et al* (21) reported four ACLF/death patients with an HEV genotype 3 infection, and HEV should be considered an acute insult in the acute decompensation of cirrhosis and ACLF. The present study also observed ACLF, which was associated with HEV genotype 3b and alcohol-induced acute insult and chronic liver disease in a patient in Japan.

Barbosa *et al* (21) reported that 50% of the patients with HEV genotype 3 infection were male, the median age was 63 years (range, 51-76 years), and the median ALT level at presentation was 2,486 U/l (range, 1,146-3,134 U/l) in the majority of cases of HEV-related ACLF. Among the causes of cirrhosis in these 4 patients, in 1 and 3 patients, this was caused by non-alcoholic steatohepatitis and alcohol-use, respectively (21). The data of the patient described in the present study were in agreement with this previous report (21).

The peak ALT level was 1,067 IU/l in the present case. High ALT levels may provide an indication for the diagnosis of acute HEV infection (21,23). Barbosa *et al* (21) also used ribavirin in 3 patients; however, in the present study, ribavirin could not be used due to renal dysfunction. In general, pregnancy, severe anemia or renal dysfunction prohibit the use of ribavirin.

Although the present study did not measure HEV viral loads, HEV RNA became undetectable on day 34 in the present case (Table II). It appears to be more beneficial for patients to eradicate the hepatitis virus causing the hepatitis. More effective anti-HEV drugs need to be developed for severe HEV infection, particularly, in pregnant females (24). HEV-infected patients with cirrhosis with or without HBV infection may develop ACLF, which is associated with a high mortality rate (~70%) (25-27).

The patient in the present study had consumed horse sashimi approximately 1 month prior to the onset of his symptoms, such as abdominal distention, loss of appetite, epigastric pain and dark urine (jaundice). It has been reported that anti-HEV IgG antibody and/or HEV RNA are positive in workhorses or horses in Egypt (28), China (29), the Netherlands (30), Bulgaria (31) and Germany (32). However, since whether horses play a role in the transmission of HEV remains unknown, further studies are warranted in this regard.

HEV has been reported to be the most common cause of infection in 95 (46.1%) of 206 patients with acute sporadic viral hepatitis and 60 (67.4%) of 89 patients with ACLF in India, an endemic country of HEV genotype 1 infection (11). Lim *et al* (33) reported that a 59-year-old Caucasian male acquired HEV infection and fatal hepatic decompensated alcohol-associated liver cirrhosis in the United Kingdom, where the HEV genotype 3 is a major genotype. Fantilli *et al* (34) reported that HEV genotype 3 infection in a patient with alcohol-associated liver disease developed ACLF in Argentina.

To the best of our knowledge, there are two reported cases from Japan, where patients with primary biliary cholangitis were infected with HEV genotype 3b (35,36). Of these 2 patients, 1 patient succumbed due to the rupture of hepatocellular carcinoma (36). Another study reported that a 49-year-old male with excessive alcohol consumption and acute HEV genotype 4 infection developed acute liver failure (37). Thus, HEV genotype 3 or HEV genotype 4 is also a key acute insult in ACLF. Of interest, among patients with alcohol-associated liver cirrhosis, a higher prevalence of anti-HEV IgG has been observed in Poland (38) and Argentina (39).

Pegylated interferon-α with or without ribavirin may play a role in eradicating HEV in some patients, although pegylated interferon-α has more side-effects (40). Several drugs, such as interferon-λ, sofosbuvir, azithromycin and ritonavir, exert anti-viral effects on HEV replication *in vitro* (41,42). Further studies are required for the development of drugs against HEV infection.

In conclusion, careful attention should be paid to viral hepatitis, including hepatitis E, in patients with alcohol-associated liver disease. Further research and the development of novel drugs for HEV infection are required for the prevention of severe HEV infections.

## Figures and Tables

**Table I:** Laboratory data of the patient upon admission (on day 0).

Item	Values	Item	Values	Item	Values
WBC	5,600 /µl	**AST**	**708 IU/l**	NH_3_	43 µg/dl
RBC	479x10^4^/µl	**ALT**	**1,067 IU/l**	anti-HIV	Negative
Hemoglobin	14.5 g/dl	**LDH**	**410 IU/l**	HBsAg	Negative
Platelets	132x10^3^/µl	**ALP**	**491 IU/l**	**anti-HBc**	**Positive**
Neutrophils	70.1%	**γ-GTP**	**443 IU/l**	anti-HBc IgM	Negative
Basophils	0.4%	CPK	66 U/ml	anti-HCV	Negative
Eosinophils	1.1%	**T. Bil**	**6.6 mg/dl**	anti-HAV IgM	Negative
Monocytes	7.7%	**D. Bil**	**5.3 mg/dl**	**anti-HEV IgA**	**Positive**
Lymphocytes	20.6%	TP	6.5 g/dl	**HEV RNA**	**Positive**
PT, INR	94%, 1.04	Albumin	3.4 g/dl	ANA	Negative
T. CHO	130 mg/dl	**BUN**	**48.7 mg/dl**	AMA M2	Negative
TG	194 mg/dl	**Creatinine**	**2.2 mg/dl**	IgG	1,391 mg/dl
Glucose	161 mg/dl	**eGFR**	**24.2 ml/min/1.73 m^2^**	IgA	336 mg/dl
**HbA1c**	**8.6%**	**CRP**	**3.4 mg/dl**	IgM	143 mg/dl

Non-bold values indicate within the normal limits. WBC, white blood cell count; RBC, red blood cell count; PT, prothrombin time; INR, international normalized ratio; T. CHO, total cholesterol; TG, triglyceride; HbA1c, hemoglobin A1c; AST, aspartate aminotransferase; ALT, alanine aminotransferase; LDH, lactate dehydrogenase; ALP, alkaline phosphatase; γ-GTP, γ-glutamyl transpeptidase; T. Bil, total bilirubin; D. Bil, direct bilirubin; TP, total protein; BUN, blood urea nitrogen; eGFR, estimated glomerular filtration rate; CRP, C-reactive protein; CPK, creatine phosphokinase; NH_3_, ammonia; anti-HIV, anti-human immunodeficiency virus antibody; HBsAg, hepatitis B surface antigen; anti-HBc, anti-hepatitis B core antibody; Ig, immunoglobulin; anti-HCV, anti-hepatitis C virus antibody; anti-HAV, anti-hepatitis A virus antibody; anti-HEV, anti-hepatitis E virus antibody; GT, genotype; ANA, anti-nuclear antibody; AMA M2, anti-mitochondrial M2 antibody.

**Table II:** Changes in biochemical and virological parameters of the patient following admission.

Day	AST (IU/l)	ALT (IU/l)	T. Bil (mg/dl)	Cre (mg/dl)	Anti-HEV IgG	COI	Anti-HEV IgM	COI	Anti-HEV IgA	COI	HEV RNA
0	708	1,067	6.6	2.2	1.724	**+**	2.728	**+**	1.963	**+**	+
1	533	849	6.2	1.9	1.766	**+**	2.517	**+**	2.135	**+**	+
5	145	315	10.3	1.4							
7	181	270	15.4	1.3							
8	192	247	15.6	1.2							
11	273	294	22.7	1.3							
13	287	320	25.9	1.4	2.680	**+**	2.099	**+**	2.279	**+**	+
14	246	285	24.2	1.4	2.617	**+**	1.901	**+**	2.264	**+**	+
15	221	277	26.3	1.4	2.630	**+**	1.838	**+**	2.218	**+**	+
18	92	141	24.7	1.5	2.616	**+**	1.777	**+**	2.203	**+**	+
20	68	97	23.5	1.6	2.583	**+**	1.741	**+**	2.207	**+**	+
22	66	72	22.3	1.6	2.502	**+**	1.718	**+**	2.236	**+**	+
25	62	56	18.1	1.5	2.476	**+**	1.748	**+**	2.236	**+**	+
26	54	48	15.2	1.5	2.482	**+**	1.688	**+**	2.163	**+**	+
27	55	47	13.7	1.4	2.562	**+**	1.679	**+**	2.053	**+**	+
29	69	51	13.1	1.4	2.562	**+**	1.720	**+**	2.061	**+**	+
32	58	49	9.3	1.3	2.582	**+**	1.595	**+**	1.848	**+**	+
34	62	54	8.2	1.4	2.628	**+**	1.577	**+**	1.840	**+**	-
36	56	52	7.1	1.4	2.562	**+**	1.420	**+**	1.771	**+**	-
39	60	61	6.5	1.4	2.514	**+**	1.508	**+**	1.845	**+**	-
41	43	49	5.1	1.4	2.556	**+**	1.440	**+**	1.710	**+**	-
43	41	48	4.3	1.3	2.814	**+**	1.453	**+**	1.672	**+**	-
48	51	48	3.7	1.3	2.812	**+**	1.456	**+**	1.564	**+**	-
61	37	37	2.3	1.3	2.751	**+**	1.592	**+**	1.265	**+**	-
83	30	32	1.4	1.2	2.770	**+**	1.026	**+**	0.992	**+**	-

**+** Symbols shown in bold font indicate more than the COI. The OD values of 0.152, 0.440 and 0.642 were used as the COI for the anti-HEV IgG, anti-HEV IgM and anti-HEV IgA, respectively (18). Day 0, day of admission; day 43, day of hospital discharge; COI, cut-off index; AST, aspartate aminotransferase; ALT, alanine aminotransferase; T. Bil, total bilirubin; Cre, creatinine; Anti-HEV, anti-hepatitis E virus antibody; Ig, immunoglobulin; +, positive; -, negative.

## Data Availability

The datasets used and/or analyzed during the current study are available from the corresponding author on reasonable request.
